# Modeling the Public Health Impact of Improved Antiretroviral Therapy Restart Patterns Among People with HIV Who Have Discontinued Treatment

**DOI:** 10.36469/001c.159112

**Published:** 2026-03-26

**Authors:** Hansel E. Tookes, Cillian Copeland, Uche Mordi, James Jarrett, Rui Martins, Mark Connolly, Patrick S. Sullivan

**Affiliations:** 1 Department of Medicine University of Miami Miller School of Medicine, Miami, Florida, USA; 2 Global Market Access Solutions Sàrl, Chardonne, Switzerland; 3 J.E. Cairnes School of Business and Economics, University of Galway, Galway, Ireland; 4 Gilead Sciences, Inc. Foster City, California, USA; 5 Gilead Sciences Europe Ltd. Uxbridge, UK; 6 GZW Global Health Department University of Groningen, University Medical Center Groningen, Groningen, The Netherlands; 7 Global Market Access Solutions Sàrl, Chandonne, Switzerland; 8 Department of Epidemiology Rollins School of Public Health, Emory University

**Keywords:** HIV, antiretroviral therapy, rapid restart ART, economic evaluation, viral suppression, retention in care

## Abstract

**Background:**

Antiretroviral therapy (ART) has become a cornerstone of human immunodeficiency virus (HIV) management. However, a challenge in HIV care and policy is ensuring individuals remain engaged in care and on treatment over time. Discontinuation of ART is common for various reasons, and prolonged treatment interruptions can lead to worse health outcomes at the individual level and increased HIV transmissions at the public health level.

**Objective:**

A cost-consequence analysis was conducted to evaluate the economic and public health impact of reducing the interval to ART restart among people with HIV (PWH) who have disengaged from care.

**Methods:**

A state transition disease model was developed to calculate the economic benefits from improving treatment restart patterns from a United States healthcare payer perspective. Two hypothetical cohorts of 1000 PWH who discontinued ART were compared: a standard-of-care cohort where restart occurs 32 weeks after discontinuation, and a comparator cohort exploring the impact of reducing the time between ART discontinuation and restart to 12 weeks. Individuals were assigned to CD4-related health states, and rates of viral suppression were considered. Four outcomes, ART costs, CD4 health state costs, CD4-related mortality, and new HIV transmissions were calculated over a three-year time horizon. Cost savings from averted HIV cases were valued based on the lifetime excess healthcare costs for a PWH.

**Results:**

Increasing the proportion of individuals restarting ART and reducing time to restart was estimated to avert 88 HIV transmissions. This corresponds to a number needed to treat, defined as the number of PWH who would need to experience the earlier restart pattern of the comparator cohort, of 11 to avoid one new transmission, and $101 083 857 lifetime cost savings. Cost savings attributable to improved CD4 counts in the cohort were also found.

**Conclusion:**

Enhancing ART restart patterns improves health and provides considerable cost savings by improving individuals’ CD4 counts and reducing new HIV transmissions from people who are viremic. Effective policies to promote care engagement and treatment adherence are predicted to improve the health of PWH and reduce new HIV cases.

## BACKGROUND

Widespread access to antiretroviral therapy (ART) has dramatically changed human immunodeficiency virus (HIV) management, enabling millions of people globally to live with HIV as a manageable chronic condition. In the United States (US), an estimated 1.2 million people are living with HIV; however, only approximately 66% achieve durable viral suppression, which is essential for preventing disease progression and onward transmission.^1^ Discontinuation of ART and prolonged treatment interruptions remain a considerable public health concern.^2^ The reasons for stopping ART are multifactorial and can include individual and structural barriers, psychological challenges, or financial and logistical issues.^3^ Psychosocial factors including mental health disorders, substance use disorders, stigma, discrimination, and lack of support services can disrupt the HIV care continuum.^3^ Studies indicate that, in the US, approximately 5.6% of adults receiving HIV care discontinue ART at some point after starting treatment, although this can vary considerably depending on factors such as regimen or funding source. Discontinuation rates globally have been reported at 22.3%, although cross-country comparisons can be challenging due to differences in study design, population and definition of discontinuation.^4^ As treatment programs scale up and treatment eligibility broadens, understanding the drivers of ART restart among people living with HIV (PWH) who have discontinued their ART has become an essential component of our public health response.^5^ Although factors associated with ART discontinuation have been extensively studied, the dynamics of ART restart, including the proportion of PWH who restart and the timepoints at which restart occurs, has historically had comparably limited research.^6^ There is now increasing focus on the importance of programs to improve reengagement with care and reinitiation of treatment,^7^ although few studies have explored the public health and economic outcomes of these programs.

Prolonged treatment interruptions are associated with HIV viremia, decreased CD4 cell counts, increased risk of opportunistic infections, HIV disease progression, lower likelihood of resuppression, and higher rates of antiretroviral resistance.^8–12^ HIV viremia not only undermines the individual’s health but significantly increases the risk of HIV transmission.^12,13^ Large cohort and randomized controlled trial data confirm that sexual transmission of HIV is prevented when viral loads are suppressed below 200 copies/mL with sustained ART adherence.^12,14–17^ Conversely, ART interruptions or intermittent adherence result in higher community viral loads, which increase HIV transmissions.^18^ Most new HIV cases in the US are attributable to faults in the HIV care continuum: undiagnosed HIV infections, failures of retention in care, or ART disengagement.^19,20^ Retention in care and reengagement with care in this context represent two similar but distinct concepts. Retention in care relates to ongoing regular engagement with HIV services, while reengagement relates to the return to HIV care after a period of treatment interruption.^21^

Reducing or eliminating HIV transmissions imparts both clinical and economic benefits. Each new HIV case imposes substantial costs on individuals and on the wider healthcare system; the average lifetime excess healthcare costs of HIV are estimated at over $1100 000.^22^ Frequent disengagement from care creates operational costs for health systems due to the significant resources and counseling required to reengage patients.^23^ Moreover, preventable HIV transmissions from people who lose viral suppression after treatment discontinuation represent an opportunity to leverage the effectiveness of ART as prevention.^24^ Policies and programs for people who have discontinued HIV care that support faster reengagement in care and ART restart could, therefore, yield significant downstream savings, by improving individual health, and by reducing onward HIV transmissions and associated treatment costs. At an individual level, prolonged ART interruptions result in accelerated HIV disease progression, leading to increased healthcare utilization, and costly inpatient admissions caused by opportunistic infections and comorbidities.^25,26^

Despite the implications of greater disease burden for individuals and society after discontinuation, there have been limited attempts to understand the economic value of improving ART restart rates and/or shortening the time between ART discontinuation and restarting. We conducted a cost-consequence analysis to assess the potential gains associated with improved ART restart patterns in the US context. Specifically, we evaluated how increasing the proportion of PWH who restart ART after interruption and reducing the time they spend with viremia following disengagement impact HIV incidence and overall healthcare expenditures. This represents a novel study approach by evaluating the cost consequences attributable to reducing the time between treatment discontinuation and reinitiation within the US context.

## METHODS

### Model Structure

A state transition disease model was developed to conduct a cost-consequence analysis of improving ART restart patterns among a hypothetical cohort of PWH who newly discontinue ART using a US healthcare payer perspective. This involved simulating 1000 individuals who have discontinued ART and tracked their CD4 counts, viral suppression, mortality, and HIV transmissions each week over three years under two restart scenarios. A cost-consequence analysis was selected to provide policy-relevant evidence on the consequences of improving ART restart patterns. As a range of initiatives can influence treatment restart, including both treatment-related interventions and broader structural policies, this framework was considered most appropriate as it facilitates the presentation of disaggregated clinical and economic outcomes.

A three-year time horizon was chosen to investigate the short-term consequences of different restart patterns, and half-cycle correction was applied.^27^ A shorter time horizon was selected as the model focuses on the economic impact of the first treatment restart following discontinuation rather than long-term patterns of repeated discontinuations and restarts. A 1-week cycle length was selected to capture the necessary granularity in changes in treatment status, viral suppression, and CD4 count that occur following ART discontinuation and restart, and to ensure methodological consistency with prior modeling of rapid ART initiation.^28^ A Consolidated Health Economic Evaluation Reporting Standards (CHEERS) checklist^29^ was completed to ensure comprehensive and transparent reporting of all relevant methods and results in this study, and is outlined in **Supplementary Appendix 1**.

The model structure was developed around key health states defined by CD4 count and viral suppression, in line with standard modeling approaches in HIV,^28,30–32^ and builds upon a published analysis investigating the economic impact of improving rapid start of ART among newly diagnosed PWH.^28^ Individuals were distributed across five possible CD4-related health states (<50, 50-199, 200-349, 350-499, ≥500) and simultaneously categorized based on achievement of viral suppression or not (with viral suppression defined as viral load <50 copies/mL). Two sets of transition probabilities were used to model movement between the five CD4 health states, with individuals able to transition only to adjacent CD4 health states each cycle. For those on treatment, treatment specific transitions were sourced from a published evaluation by the National Institute of Health and Care Excellence (NICE)^30^; off-treatment transitions were informed by a published natural history study.^3,33^ Each CD4 state was assigned a per-cycle probability of death to capture HIV-related mortality.^34^ The proportion of PWH achieving viral suppression on ART was sourced from published clinical data.^35,36^ The specific transition probabilities are provided in **Supplementary Appendix 2**.

### Transmission Calculations

A key benefit of ART is the reduction in risk of onward transmission of HIV.^6^ This benefit was captured in the model by accounting for onward transmission among individuals who are not virally suppressed (ie, those with viral load ≥50 copies/mL). New transmissions were calculated by combining per-risk-act transmission probabilities among viremic individuals, with average risk acts occurring per week.^37,38^ Separate transmission probabilities were sourced for three core groups: heterosexual men, heterosexual women, and men who have sex with men (MSM), and then combined to generate a weighted estimate based on the relative size of each group. These weighted estimates were sourced from the breakdown of the prevalent population of PWH reported by the US Centers for Disease Control and Prevention, and consisted of 10.1%, 22.8%, and 67.1% heterosexual men, heterosexual women, and MSM, respectively.^39^ Combining per-act transmission probabilities, risk acts per week, and the relative size of each subgroup resulted in a weighted per-cycle transmission probability of 0.00059 and 0.00791 for individuals not virally suppressed on treatment and individuals not virally suppressed off treatment, respectively. A detailed breakdown of transmission-related parameters are provided in **Supplementary Appendix 2**.

### Cost Inputs

A range of costs were applied in the model. Each CD4 health state was assigned a weekly cost, reflecting costs such as opportunistic infection prophylaxis, inpatient and outpatient utilization, and emergency department visits.^40^ A weekly ART cost was applied, based on mean prescription costs from an observational study evaluating healthcare costs among PWH.^22^ This same study reports the lifetime excess healthcare cost of a PWH compared with an individual without HIV, which was used to value each onward transmission occurring in the model.^22^ This lifetime healthcare cost was applied to capture the full benefit of each transmission avoided within the three-year time horizon.

All costs were inflated to 2025 values using relevant US indices^41^ and were discounted at an annual rate of 3%, in line with guidelines for US economic evaluations.^42,43^ **Table 1** presents the healthcare cost inputs used in the analysis with details of the original costs outlined in **Supplementary Appendix 2**.

**Table 1.** Healthcare Cost Inputs Used to Model the Economic Consequences of a New HIV Infection in the US

**Input**	**Inflated Cost Applied in Model, $**
Lifetime cost of HIV infection	1164 171
ART costs (per week)	871.96
Health state costs (per week)	
CD4 <50	966.62
CD4 50-199	433.83
CD4 200-349	232.48
CD4 350-500	164.67
CD4 >500	122.44

Abbreviation: ART, antiretroviral therapy; CD4, cluster of differentiation 4.

### Cohort Definition and Calculations

Two cohorts were compared in the analysis. In the standard-of-care (SoC) cohort, the rates and timepoint of ART restart reflected current observed patterns, and in the “rapid restart” cohort, improved restart was assumed. All individuals were assumed to enter the model in the cycle at which they discontinued their ART, and their baseline CD4 count categories were distributed in line with a cohort of treatment experienced PWH, as shown in **Table 2.**

**Table 2.** Distribution of CD4 Counts at the Model Start in PWH Who Have Discontinued ART

**CD4 Count**	**Baseline Proportion, %**
CD4 <50	0.00
CD4 50-199	1.65
CD4 200-349	7.50
CD4 350-499	16.70
CD4 ≥500	74.15

Abbreviation: CD4, cluster of differentiation 4.

Source: Molina et al (2018),^44^ Daar et al (2018).^45^

All individuals were assumed to be receiving the benefits of ART at model entry, in terms of both improved CD4 count transitions and high rates of viral suppression. Treatment benefit from ART was assumed to wane over three weeks or the first three model cycles after ART discontinuation, based on the observed time to viral rebound following ART discontinuation which has been reported at between 16 to 22 days.^10,11^ Following this waning period, individuals were assumed to be viremic and experience CD4 transitions in line with an untreated cohort.^34^

Treatment restart was captured through two components: the proportion of individuals restarting treatment and the timepoint at which restart occurred. These inputs were sourced from a published analysis of ART reinitiation patterns among Medicaid recipients reporting that 56% of individuals reinitiated ART 8.2 months (median) after ART discontinuation.^46^ Therefore, in the base case, we have modeled 56% of individuals in the SoC cohort to restart ART 8 months following discontinuation (ie, 32 model cycles). For the rapid restart cohort, we assumed that 56% of individuals restarted ART; however, reinitiation was assumed to occur at three months (ie, 12 weekly model cycles). This assumption reflects individuals restarting ART at their first point of reengagement three months after discontinuation.^21,47^ **Table 3** outlines the composition of each cohort.

**Table 3.** Model Inputs Informing the Proportion of Individuals Restarting ART and Its Timing in the Standard of Care vs Rapid Restart Cohorts

	**SoC Cohort**	**Rapid Restart Cohort**
Proportion restarting ART, %	56	56
Timepoint of ART restart (cycles)^a^	32	12

Abbreviations: ART, antiretroviral therapy; SoC: standard of care.

^a^Time from discontinuation to ART restart. Based on a 1-week cycle length.

### Sensitivity Analysis

A range of scenario analyses were conducted to test the impact of altering model assumptions related to cohort composition, time horizon, and other relevant inputs. Key scenarios are described below, while **Supplementary Appendix 3** contains additional scenarios alongside a probabilistic sensitivity analysis (PSA):

- The proportion of individuals restarting treatment was increased by 20% in the rapid restart cohort.
- The rapid restart cohort included a 20% increase in the proportion of individuals restarting treatment while keeping a constant restart timepoint of 12 weeks in both cohorts.
- The base case restart timepoint in the SoC cohort was updated to 52 weeks, compared with 12 weeks in the rapid restart cohort.
- The average probability of a viremic individual transmitting HIV was decreased by 20%.
- The average lifetime cost of averting 1 HIV infection was decreased by 20%.

## RESULTS

### Base Case Results

The base case analysis explored the impact of reducing the time between treatment discontinuation and reinitiation by 5 months in the rapid restart cohort. Over the three-year model horizon, 0.2 deaths were averted in the cohort of 1000, corresponding to a number needed to treat of 5427 to avoid one death. The earlier timepoint of ART restart led to individuals in the rapid restart cohort spending more time on treatment, resulting in an increase in ART costs of $9 655 702. However, because this additional time on treatment improved CD4 count, CD4 health state cost savings of $165 296 were estimated. Due to more individuals initiating treatment and subsequently achieving viral suppression in the rapid restart cohort, 88 onward transmissions were averted, with a number needed to treat of 11 to avoid one new transmission. These 88 averted infections led to lifetime cost savings of $101 083 857. Deterministic results for the base case analysis are outlined in **Table 4**. Probabilistic results, including 95% confidence intervals, are provided in **Supplementary Appendix 3**, and reflect a PSA with 1000 iterations.

**Table 4.** Results Comparing Standard of Care and Rapid Restart Cohorts After ART Discontinuation (Discounted)

	**SoC Cohort**	**Rapid Restart Cohort** ^a^	**Incremental^b^**
Deaths, n	9	9	−0.2
Onward transmissions, n	662	574	−88
ART costs, $	57 419 420	67 075 122	9 655 702
CD4 health state costs, $	24 091 997	23 926 701	−165 296
Cost of onward transmissions, $	742 301 393	641 217 536	−101 083 857

Abbreviations: ART, antiretroviral therapy; CD4, cluster of differentiation 4; SoC, standard of care.

^a^Fifty-six percent of PWH restarted ART after discontinuation in both cohorts, with restart occurring at 32 weeks in the SoC cohort and at 12 weeks in the rapid restart cohort.

^b^Negative incremental costs denote cost savings for the rapid restart cohort.

### Scenario Analysis

Results of the scenario analyses are outlined in **Table 5**. The most impactful scenario results from combining the base case assumptions of earlier treatment restart with a higher proportion of individuals restarting, while conservative scenarios exploring reductions in transmission probability and lifetime HIV cost show lower net savings compared with the base case. Further scenario analyses as well as results of the PSA are provided in **Supplementary Appendix 3**.

**Table 5.** Results of Scenario Analyses Comparing Standard of Care and Rapid Restart Cohorts After ART Discontinuation (Discounted)

**Scenario**	**Inc. ART Costs, $**	**Inc. CD4 HS Costs, $**	**Inc. HIV Transmission**	**Inc. Costs from Averted Transmissions, $**	**Net Inc. Costs^a^, $**
Base case analysis	9 655 702	−165 296	−88	−101 083 857	−91 593 451
20% increase in restart proportion	23 070 726	−533 147	−214	−241 647 730	−219 110 151
Both cohorts restart at 12 weeks, 20% increase in restart proportion	13 415 024	−367 851	−127	−140 563 873	−127 516 699
SoC restart timepoint at 52 weeks	19 201 717	−398 271	−176	−200 919 479	−182 116 034
20% decrease in transmission probability	9 655 702	−165 296	−70	−80 867 086	−71 376 680
20% decrease in lifetime HIV cost	9 655 702	−165 296	−88	−80 867 086	−71 376 680

Abbreviations: ART, antiretroviral therapy; CD4, cluster of differentiation 4; HS, health state; Inc, incremental; SoC, standard of care.

^a^Negative incremental costs denote cost savings for the rapid restart cohort.

## DISCUSSION

### Key Findings and Interpretation

Improving treatment restart among PWH who have discontinued ART is estimated to result in individual benefits from improved disease outcomes and wider public health benefits from reducing onward transmissions. There is increasing focus on the importance of ensuring PWH who discontinue ART are restarted on treatment as soon as possible to maintain their health; prolonged periods of disengagement create a range of risks for PWH related to disease progression, mortality, and onward transmission.^7,48,49^ However, there is limited research exploring the economic benefits associated with improvements to treatment restart patterns.^50^ Our results reveal that decreasing the time between treatment discontinuation and reinitiation is estimated to generate significant savings. The savings in our model came from 88 averted new transmissions, corresponding to cumulative cost savings of $101 083 857 per 1000 individuals with timely restarts after discontinuing ART. We explored a wide range of scenario analyses to understand how changing assumptions related to restart patterns, cohort composition, and key inputs impacted results. In general, although the magnitude of results varied, all scenarios illustrated that improving treatment restart leads to significant economic savings, while the PSA showed that the rapid restart cohort remained cost saving in all iterations. The output of the sensitivity analysis is particularly pertinent in this population, as data focusing specifically on individuals who have discontinued or interrupted treatment is sparse, therefore creating challenges in validating output when modeling this cohort.

The primary driver of model results relates to the probability and cost of onward HIV transmissions, as demonstrated by the change in outcomes when these parameters were varied in scenario analyses (**Table 5**). The number of averted onward HIV transmissions are sensitive to both the selected restart timepoint and the proportion of individuals restarting treatment. This is particularly important given the model structure, which assumes a single instance of treatment restart. After this timepoint, individuals who do not restart remain off treatment for the duration of the model horizon, resulting in progressively higher numbers of onward transmissions. The scenarios presented in **Table 5** focus on these key drivers and, while the rapid restart cohort remained cost saving across all scenarios, the magnitude of these savings varied considerably.

### Limitations

There are several limitations to this analysis, primarily driven by the limited data availability on individuals who restart ART after discontinuation or prolonged treatment interruption. Accurately characterizing the baseline population, in particular with regard to CD4 count, is challenging. The base case analysis uses a CD4 distribution reflective of a treatment experienced cohort,^44,45^ which may overestimate CD4 levels at the point of discontinuation and underestimate mortality risk. As the base case values assume individuals have high CD4 count at baseline, the incremental impact of rapid restart in improving CD4 count is relatively minor, which subsequently translates to both a modest mortality benefit (as mortality is linked to CD4 count) and minor health state cost savings As demonstrated in the scenario analysis in **Supplementary Appendix 3**, using lower baseline CD4 counts yields even greater benefits from improved restart patterns by transitioning individuals to higher CD4 states and leading to a greater mortality benefit and higher health state cost savings.^51^

Model outcomes are also substantially influenced by the estimated number of averted HIV transmissions. The base case analysis generated transmission rates by combining per-cycle risk acts with per-act transmission probabilities by subgroup; however, these rates may not fully capture the various factors influencing onward transmission such as variation in partner susceptibility or use of pre-exposure prophylaxis (PrEP). Additionally, the model assumes constant risk behavior, which may not capture the fluctuation in risk behaviors over time relating to different lifestyle factors and events.^52^ Using more conservative transmission parameters reduces overall cost savings however improving restart patterns remains a cost-saving intervention (see **Supplementary Appendix 3**).^53^ Similar trends are observed among the other scenario analyses, which explore decreases to both transmission probabilities and lifetime healthcare costs from averted HIV cases.

This analysis adopted a simplified structure by considering only a single instance of treatment restart. In practice, individuals may experience multiple treatment interruptions, which can lead to further onward transmission as well as increased mortality; therefore, this represents an important limitation of the model.^54^ Repeated treatment interruptions may also lead to the development of resistance, thereby reducing the efficacy of treatments.^55,56^ As such, interventions that improve treatment restart may also help prevent future interruptions, potentially yielding even greater benefits than those captured in this model. Finally, while this analysis evaluates the impact of a hypothetical intervention aimed at improving restart patterns, it does not incorporate the cost of implementing such an intervention, which would reduce the overall cost savings reported. Consequently, the results presented here may be considered an upper estimate of potential cost savings in the absence of implementation costs.

A limitation of this study is the use of aggregate data that is assumed to be representative of the US treatment landscape. There is a high degree of variation in treatment and broader HIV infrastructure across states.^57–59^ Variation in factors such as enrollment criteria, types and magnitude of services covered, medication formularies, and benefit limits can create disparities in treatment retention across US states.^46^ Geographical disparities in care retention are well documented in the US, with southern states typically experiencing lower retention than other areas.^57,60^ These disparities can also be driven by funding type, and differences in treatment retention patterns have been observed between individuals covered by commercial insurance, Medicare, Medicaid, or the Ryan White HIV/AIDS Program.^21,46,61^ Based on these differences, it is likely that the benefit of new interventions promoting improved ART restart will vary across individual states. However, in states where retention in care is typically low, improving ART restart patterns will likely generate even greater benefits than were observed in this analysis. Similarly, implementation of programs to improve treatment restart may yield greater benefits in healthcare programs with higher unmet need and an important area for future research is to adapt this analysis to focus on specific subgroups, whether defined by state or funding source (eg, Medicaid).

### Implications for Policy and Practice

At the healthcare system level, modeling studies from high-income countries indicate that consistent ART use yields significant cost savings by preventing new HIV cases, reducing hospitalizations, and maintaining workforce participation among PWH.^25^ When PWH discontinue therapy, not only do their healthcare costs climb, but each new cycle of onward HIV transmissions sets off an exponential increase in lifetime treatment expenses for additional individuals.^26,62^ Thus, lower rates of retention in care undermine the cost-effectiveness and fiscal sustainability of HIV programs (eg, Ryan White HIV/AIDS Program), and ART persistence is a strategic economic priority.

A range of interventions and strategies have been explored to support ART adherence and reduce treatment interruptions. These include robust psychosocial support, regular counseling, mental health services, and peer support interventions, all of which have been shown to improve retention to care and medication adherence.^63,64^ Addressing social determinants of health, such as transportation assistance, stable housing, and food support, also plays a critical role in reducing structural and individual barriers to ongoing treatment engagement.^3,64^ Simplification of ART regimens (eg, once-daily, fixed-dose combinations, long-acting therapies), patient education, and close follow-up with proactive problem-solving for side effects or life changes are proven methods for supporting ART adherence.^65–67^ Importantly, fostering a nonjudgmental, stigma-free care environment and combating discrimination further encourages individuals to remain in care and adhere to medication.^68^ Collective use of these strategies helps ensure sustained viral suppression, improves individual health outcomes, and curtails HIV transmission within communities.

Lifelong ART is vital for both individual health and ending the HIV epidemic^69,70^; this reality is recognized in global health initiatives like UNAIDS 95-95-95 targets. Sustained viral suppression through continuous ART use prevents HIV transmission, reduces individual morbidity and mortality, and offers considerable economic returns by averting new infections and promoting productivity.^14,25^ The evidence from population-level and economic studies underscores that investments in treatment retention strategies are both clinically warranted and cost-effective.^25,62^ As the HIV treatment–eligible population expands and ART coverage grows, addressing the factors influencing ART discontinuation will be critical. Innovative and patient-centered approaches to ART retention promise not only to improve the lives of PWH but also to advance public health goals of ending the HIV epidemic.^71^

## CONCLUSION

We conducted a cost-consequence analysis to evaluate the economic impact from improving patterns of ART restart among a hypothetical cohort of PWH following initial discontinuation of ART. Shortening the time to ART reinitiation leads to higher ART costs, because more individuals are on ART; however, these costs are offset by savings from both improved CD4 count and averted HIV transmissions. A modest mortality benefit was found from the associated improvements in CD4 count, which reflects the optimistic CD4 count assumed for the population at baseline and helps explain the high number needed to treat observed for mortality. Overall, improvements in ART restart patterns translated to net cost savings of over $91 million. This demonstrates that new policies aimed at improving retention in care or facilitating return to care, such as reengagement pathways, community supports or psychosocial interventions, can yield considerable clinical and economic returns, reinforcing ART restart as an integral component of the HIV care continuum.

### Disclosures

C.C., R.M., and M.C. are employees of Global Market Access Solutions, who were paid consultants to Gilead. J.J. and U.M. are employees of Gilead. P.S.S. has received research grants and speaker fees from Gilead Sciences; he has also received research grants from Merck and ViiV Healthcare. H.E.T. has received grants from Gilead Sciences and ViiV Healthcare.

## Supplementary Material

Online Supplementary Material
