# Supplementary material for: Modeling the Public Health Impact of Improved Antiretroviral Therapy Restart Patterns Among People with HIV Who Have Discontinued Treatment
Source: J Health Econ Outcomes Res. 2026 Mar 26;13(1):103–10. doi: 10.36469/001c.159112 (PMC13032768; doi:10.36469/001c.159112)
Supplement: Online Supplementary Material [file jheor_2026_13_1_159112_337071.pdf]

Journal of Health Economics and Outcomes Research

## Online Supplementary Material

Modeling the Public Health Impact of Improved Antiretroviral Therapy Restart Patterns Among People with HIV Who Have Discontinued Treatment. *JHEOR*. 2026;13(1):103-110. doi:10.36469/jheor.2026.159112

**Appendix 1: CHEERS Checklist**

**Appendix 2: Additional Model Inputs**

**Table S1: Transition Probabilities – On Treatment, Viral Suppression (Weeks 0-48)**

**Table S2: Transition Probabilities – On Treatment, No Viral Suppression (Weeks 0-48)**

**Table S3: Transition Probabilities – On Treatment, Viral Suppression (Weeks 49+)**

**Table S4: Transition Probabilities – On Treatment, No Viral Suppression (Weeks 49+)**

**Table S5: Transition Probabilities – Off Treatment**

**Table S6: Inputs Used to Model HIV Transmissions Arising from Heterosexual Men and Women, and Men Who Have Sex with Men, United States**

**Table S7: Healthcare Cost Inputs Used to Model the Economic Consequences of a New HIV Infection in the United States**

**Appendix 3: Additional Sensitivity Analysis**

**Table S8: Alternate CD4 Count Distribution**

**Table S9: Supplemental Scenario Analysis Results**

**Table S10: Probabilistic Incremental Results (Mean and 95% CI) Over 1,000 Iterations**

This supplementary material has been provided by the authors to give readers additional information about their work.

This is an open-access article distributed under the terms of the Creative Commons Attribution 4.0 International License (CCBY-4.0). View this license's legal deed at http://creativecommons.org/licenses/by/4.0 and legal code at http://creativecommons.org/licenses/by/4.0/legalcode for more information.

# Supplemental Appendix 1: CHEERS Checklist

## Consolidated Health Economics Evaluation Reporting Standards 2022 (CHEERS 2022) Statement[1]

| Section | Topic | Item No. | Guidance for Reporting | Reported in Section |
|---|---|---|---|---|
| **Title** | Title | 1 | Identify the study as an economic evaluation and specify the interventions being compared. | Provided in title |
| **Abstract** | Abstract | 2 | Provide a structured summary that highlights context, key methods, results, and alternative analyses. | Manuscript abstract |
| **Introduction** | Background and objectives | 3 | Give the context for the study, the study question, and its practical relevance for decision making in policy or practice. | Lines 2-61 |
| **Methods** | Health economic analysis plan | 4 | Indicate whether a health economic analysis plan was developed and where available. | N/A |
| | Study population | 5 | Describe characteristics of the study population (such as age range, demographics, socioeconomic, or clinical characteristics). | Lines 64-69 |
| | Setting and location | 6 | Provide relevant contextual information that may influence findings. | N/A |
| | Comparators | 7 | Describe the interventions or strategies being compared and why chosen. | Lines 135-137, 152-160 |
| | Perspective | 8 | State the perspective(s) adopted by the study and why chosen. | Line 66-67 |
| | Time horizon | 9 | State the time horizon for the study and why appropriate. | Line 74-77 |
| | Discount rate | 10 | Report the discount rate(s) and reason chosen. | Line 127-128 |
| | Selection of outcomes | 11 | Describe what outcomes were used as the measure(s) of benefit(s) and harm(s). | Lines 67-69, 94-95, 108-110, 119-125 |

| Section | Topic | Item No. | Guidance for Reporting | Reported in Section |
|---|---|---|---|---|
| Methods | Measurement of outcomes | 12 | Describe how outcomes used to capture benefit(s) and harm(s) were measured. | Line 87-95, 108-112 |
| | Valuation of outcomes | 13 | Describe the population and methods used to measure and value outcomes. | Line 94-95, 119-126 |
| | Measurement and valuation of resources and costs | 14 | Describe how costs were valued. | Line 119-126, Table 1 |
| | Currency, price date, and conversion | 15 | Report the dates of the estimated resource quantities and unit costs, plus the currency and year of conversion. | Table 1, Supplemental Appendix 2 (Table S7) |
| | Rationale and description of model | 16 | If modeling is used, describe in detail and why used. Report if the model is publicly available and where it can be accessed. | Line 64-73 |
| | Analytics and assumptions | 17 | Describe any methods for analysing or statistically transforming data, any extrapolation methods, and approaches for validating any model used. | N/A |
| | Characterizing heterogeneity | 18 | Describe any methods used for estimating how the results of the study vary for subgroups. | N/A |
| | Characterizing distributional effects | 19 | Describe how impacts are distributed across different individuals or adjustments made to reflect priority populations. | N/A |
| | Characterizing uncertainty | 20 | Describe methods to characterize any sources of uncertainty in the analysis. | Line 165-177, 198-208, Table 5, |
| | Approach to engagement with patients and others affected by the study | 21 | Describe any approaches to engage patients or service recipients, the general public, communities, or stakeholders (such as clinicians or payers) in the design of the study. | N/A |
| Results | Study parameters | 22 | Report all analytic inputs (such as values, ranges, references) including uncertainty or distributional assumptions. | Table 1-Table 4, Supplemental Appendix 2 |
| | Summary of main results | 23 | Report the mean values for the main categories of costs and outcomes of interest and summarize them in the most appropriate overall measure. | Table 5 |
| | Effect of uncertainty | 24 | Describe how uncertainty about analytic judgments, inputs, or projections affect findings. Report the effect of choice of discount rate and time horizon, if applicable. | 161-166, Table 6, Supplemental Appendix 3 |
| | Effect of engagement with patients and others affected by the study | 25 | Report on any difference patient/service recipient, general public, community, or stakeholder involvement made to the approach or findings of the study | N/A |
| Discussion | Study findings, limitations, generalisability, and current knowledge | 26 | Report key findings, limitations, ethical or equity considerations not captured, and how these could affect patients, policy, or practice. | Line 211-292 |
| Other relevant information | Source of funding | 27 | Describe how the study was funded and any role of the funder in the identification, design, conduct, and reporting of the analysis | Included in cover letter |
| | Conflicts of interest | 28 | Report authors conflicts of interest according to journal or International Committee of Medical Journal Editors requirements. | Included in cover letter |

# Supplemental Appendix 2: Additional Model Inputs

## Table S1. Transition Probabilities – On Treatment, Viral Suppression (Weeks 0-48)

| | To | | | | | |
|---|---|---|---|---|---|---|
| **Viral load*** | **<50** | | | | | |
| **CD4 cell count^** | | **<50** | **50-199** | **200-349** | **350-500** | **>500** |
| **From** | **<50** | 0.93 | 0.06 | 0.00 | 0.00 | 0.00 |
| | **50-199** | 0.02 | 0.92 | 0.06 | 0.00 | 0.00 |
| | **200-349** | 0.00 | 0.02 | 0.93 | 0.05 | 0.00 |
| | **350-500** | 0.00 | 0.00 | 0.02 | 0.93 | 0.05 |
| | **>500** | 0.00 | 0.00 | 0.00 | 0.01 | 0.99 |
| *Viral load in copies/mL ^CD4 cell count in cells/mm3 | | | | | | |

Abbreviation: CD4, cluster of differentiation 4.

**Source:** NICE TA757.[2]

## Table S2. Transition Probabilities – On Treatment, No Viral Suppression (Weeks 0-48)

| | To | | | | | |
|---|---|---|---|---|---|---|
| **Viral load*** | **≥50** | | | | | |
| **CD4 cell count^** | | **<50** | **50-199** | **200-349** | **350-500** | **>500** |
| **From** | **<50** | 0.93 | 0.06 | 0.00 | 0.00 | 0.00 |
| | **50-199** | 0.02 | 0.92 | 0.06 | 0.00 | 0.00 |
| | **200-349** | 0.00 | 0.02 | 0.93 | 0.05 | 0.00 |
| | **350-500** | 0.00 | 0.00 | 0.02 | 0.93 | 0.05 |
| | **>500** | 0.00 | 0.00 | 0.00 | 0.01 | 0.99 |
| *Viral load in copies/mL ^CD4 cell count in cells/ $mm^3$ | | | | | | |

Abbreviation: CD4, cluster of differentiation 4.

**Source:** NICE TA757.[2]

## Table S3. Transition Probabilities – On Treatment, Viral Suppression (Weeks 49+)

| | To | | | | | |
|---|---|---|---|---|---|---|
| **Viral load*** | **<50** | | | | | |
| **CD4 cell count^** | | **<50** | **50-199** | **200-349** | **350-500** | **>500** |
| **From** | **<50** | 0.88 | 0.11 | 0.00 | 0.00 | 0.00 |
| | **50-199** | 0.03 | 0.91 | 0.06 | 0.00 | 0.00 |
| | **200-349** | 0.00 | 0.03 | 0.92 | 0.05 | 0.00 |
| | **350-500** | 0.00 | 0.00 | 0.03 | 0.92 | 0.05 |
| | **>500** | 0.00 | 0.00 | 0.00 | 0.01 | 0.99 |
| *Viral load in copies/mL ^CD4 cell count in cells/ $mm^3$ | | | | | | |

Abbreviation: CD4, cluster of differentiation 4.

**Source:** NICE TA757.[2]

## Table S4. Transition Probabilities – On Treatment, No Viral Suppression (Weeks 49+)

| | To | | | | | |
|---|---|---|---|---|---|---|
| **Viral load*** | **≥50** | | | | | |
| **CD4 cell count^** | | **<50** | **50-199** | **200-349** | **350-500** | **>500** |
| **From** | **<50** | 0.88 | 0.11 | 0.00 | 0.00 | 0.00 |
| | **50-199** | 0.03 | 0.91 | 0.06 | 0.00 | 0.00 |
| | **200-349** | 0.00 | 0.03 | 0.92 | 0.05 | 0.00 |
| | **350-500** | 0.00 | 0.00 | 0.03 | 0.92 | 0.05 |
| | **>500** | 0.00 | 0.00 | 0.00 | 0.01 | 0.99 |
| *Viral load in copies/mL ^CD4 cell count in cells/mm$^3$ | | | | | | |

Abbreviation**:** CD4, cluster of differentiation 4.

**Source:** NICE TA757.[2]

## Table S5: Transition Probabilities – Off Treatment

| | To | | | | | |
|---|---|---|---|---|---|---|
| **CD4 cell count^** | | **<50** | **50-199** | **200-349** | **350-500** | **>500** |
| **From** | **<50** | 1.00 | 0 | 0 | 0 | 0 |
| | **50-199** | 0.01 | 0.99 | 0 | 0 | 0 |
| | **200-349** | 0 | 0.00 | 1.00 | 0 | 0 |
| | **350-500** | 0 | 0 | 0.01 | 0.99 | 0 |
| | **>500** | 0 | 0 | 0 | 0.00 | 1.00 |
| *Viral load in copies/mL ^CD4 cell count in cells/mm$^3$ | | | | | | |

Abbreviation**:** CD4, cluster of differentiation 4.

**Source:** Mangal et al 2017.[3]

## Table S6. Inputs Used to Model HIV Transmissions Arising from Heterosexual Men and Women, and Men Who Have Sex with Men, United States

| Input | On Treatment | Off Treatment | Source |
|---|---|---|---|
| Per-act transmission probability | | | |
| Heterosexual men | 0.0001 | 0.00159 | Supervie et al (2017)[4] |
| Heterosexual women | 0.00021 | 0.00318 | |
| MSM | 0.00134 | 0.01767 | |
| Average risk acts per week | 0.6209 | | Estrada et al (2022)[5] |
| **Average weekly transmissions** | **0.00059** | **0.00791** | **Calculation/CDC (2024)[6]** |

Abbreviations: CDC, Centers for Disease Control and Prevention, MSM, men who have sex with men.

## Table S7. Healthcare Cost Inputs Used to Model the Economic Consequences of a New HIV Infection in the United States

| Input | Original Cost | Inflated Cost (Applied in Model) |
|---|---|---|
| Lifetime cost of HIV infection | $1,117,729 (2023) | $1,164,171 |
| ART costs (per week) | $837.18 (2023) | $871.96 |
| Health state costs (per week) | | |
| CD4: <50 | $842.41 (2017) | $966.62 |
| CD4: 50-199 | $378.09 (2017) | $433.83 |
| CD4: 200-349 | $202.61 (2017) | $232.48 |
| CD4: 350-500 | $143.51 (2017) | $164.67 |
| CD4: >500 | $106.71 (2017) | $122.44 |

Abbreviations: ART, antiretroviral therapy; CD4, cluster of differentiation 4.

# Supplemental Appendix 3: Additional Sensitivity Analysis

**Table S9** details results of supplemental scenario analyses conducted in addition to those presented in the main manuscript and which had a relatively minor impact on results. These scenarios explore the following:

- The proportion of individuals restarting treatment was increased by 10% in the rapid restart cohort.
- Alternate time horizons of 1 year and 5 years were applied to base case assumptions.
- Alternate waning periods for ART efficacy of 1 week and 5 weeks were applied to base case assumptions.
- The average probability of a non-virally suppressed individual transmitting an onward HIV infection was increased by 20%.
- The average lifetime cost of averting one HIV infection was increased by 20%.
- Transmission rates were updated to reflect annual transmission rates from a 2025 publication by the Centers for Disease Control and Prevention (CDC).[7] For this scenario, transmission rates were based on the following assumption:
  - Transmission rates for "Receiving HIV care (on ART) but not virally suppressed" applied when on treatment and not virally suppressed.
  - Transmission rates for "Aware and not in care" applied for individuals not on treatment and not virally suppressed.

The final scenario explored an updated baseline CD4 count distribution based on those published for a treatment interruption population[8]. A comparison of the base case CD4 distribution and these updated values are outlined in **Table S8**.

**Table S8. Alternate CD4 Count Distribution**

| Scenario | Base Case Analysis[9,10] | Scenario[8] |
|---|---|---|
| CD4 <50 | 0.00% | 11.7% |
| CD4 50-199 | 1.65% | 22.1% |
| CD4 200-349 | 7.50% | 23.6% |
| CD4 350-499 | 16.70% | 16.4% |
| CD4 ≥500 | 74.15% | 26.3% |

Abbreviation: CD4, cluster of differentiation 4.

## Table S9. Supplemental Scenario Analysis Results

| Scenario | Inc. ART Costs | Inc. CD4 HS Costs | Inc. HIV Transmission | Inc. Costs from Averted Transmissions | Net Inc. Costs |
|---|---|---|---|---|---|
| **Base case analysis** | **$9,655,702** | **-$165,296** | **-88** | **-$101,083,857** | **-$91,593,451** |
| 10% increase in restart proportion | $16,363,214 | -$349,221 | -151 | -$171,365,793 | -$155,351,801 |
| Time horizon of 1 year | $9,635,592 | -$58,081 | -88 | -$101,388,363 | -$91,810,853 |
| Time horizon of 5 years | $9,687,839 | -$173,181 | -88 | -$100,810,390 | -$91,295,732 |
| ART waning of 1 week | $9,655,431 | -$165,792 | -88 | -$101,084,889 | -$91,595,250 |
| ART waning of 5 weeks | $9,656,419 | -$163,980 | -88 | -$101,081,713 | -$91,589,274 |
| 20% increase in transmission probability | $9,655,702 | -$165,296 | -105 | -$121,300,628 | -$111,810,222 |
| 20% increase in lifetime HIV cost | $9,655,702 | -$165,296 | -88 | -$121,300,628 | -$111,810,222 |
| Alternate source for transmission rates | $9,655,702 | -$165,296 | -12 | -$13,712,892 | -$4,222,487 |
| Alternate source for baseline CD4 distribution | $10,358,040 | -$1,844,424 | -86 | -$99,198,775 | -$90,685,160 |

Abbreviations: ART, antiretroviral therapy; CD4, cluster of differentiation 4; HS, health state; Inc., incremental.

A probabilistic sensitivity analysis was also conducted. This involved assigning each variable in the model a relevant distribution which was then sampled for 1,000 iterations. A mean probabilistic result and associated 95% confidence interval was calculated for all outcomes.

Distributions were assigned according to the standard guidelines for probabilistic analysis in health economic modeling:[11]

- **Gamma** distribution assigned for costs
- **Beta** distribution assigned for binomial probabilities
- **Dirichlet** distribution used for multinomial probabilities or proportions
- **Normal** distribution applied for remaining parameters

Mean results over 1,000 iterations, as well as the corresponding 95% confidence intervals, are outlined in **Table S10.**

**Table S10. Probabilistic Incremental Results (Mean and 95% CI) Over 1,000 Iterations**

| Category | Deterministic Results | Probabilistic Results | 95% CI |
|---|---|---|---|
| Deaths | -0.184 | -0.251 | (-0.259, -0.243) |
| Transmissions | -87.88 | -87.43 | (-88.20, -86.66) |
| ART costs | $9,655,702 | $9,693,089 | ($9,635,573, $9,750,604) |
| CD4 costs | -$165,296 | -$159,593 | (-$163,411, -$155,774) |
| Transmission costs | -$101,083,857 | -$100,568,177 | (-$101,447,047, -$99,689,307) |

Abbreviations: ART, antiretroviral therapy; CD4, cluster of differentiation 4; CI, confidence interval.

# REFERENCES


1. Husereau D, Drummond M, Augustovski F, et al. Consolidated Health Economic Evaluation Reporting Standards 2022 (CHEERS 2022) statement: updated reporting guidance for health economic evaluations. *BMC Med*. 2022;20(1):23. doi:10.1186/s12916-021-02204-0
2. National Institute of Health and Care Excellance. *Cabotegravir with rilpivirine for treating HIV-1 (TA757) - Committee Papers*. 2022. https://www.nice.org.uk/guidance/TA757
3. Mangal TD. Joint estimation of CD4+ cell progression and survival in untreated individuals with HIV-1 infection. *AIDS*. 2017;31(8):1073-1082.
4. Supervie V, Assoumou L, Breban R, et al. Risk of HIV transmission during combined ART initiation for HIV-infected persons with severe immunosuppression. *J Antimicrob Chemother*. 2017;72(11):3172-3176.
5. Estrada V, Górgolas M, Peña JA, et al. Epidemiologic and economic analysis of rapid antiretroviral therapy initiation with bictegravir/emtricitabine/tenofovir alafenamide in Spain. *Pharmacoecon Open*. 2022;6(3):415-424.
6. Centers for Disease Control and Prevention. HIV Surveillance Supplemental Report: Estimated HIV Incidence and Prevalence in the United States, 2018–2022. Accessed January 2024. https://stacks.cdc.gov/view/cdc/156513
7. Baxter A, Gopalappa C, Islam MH, et al. Updates to HIV transmission rate estimates along the HIV care continuum in the United States, 2019. *J Acquir Immune Deficiency Syndr*. 2025;99(1):47-54.
8. Trickey A, Zhang L, Rentsch CT, et al. Care interruptions and mortality among adults in Europe and North America. *AIDS*. 2024;38(10):1533-1542. doi:10.1097/qad.0000000000003924

9. Daar ES, DeJesus E, Ruane P, et al. Efficacy and safety of switching to fixed-dose bictegravir, emtricitabine, and tenofovir alafenamide from boosted protease inhibitor-based regimens in virologically suppressed adults with HIV-1: 48 week results of a randomised, open-label, multicentre, phase 3, non-inferiority trial. *Lancet HIV*. 2018;5(7):e347-e356. doi:10.1016/s2352-3018(18)30091-2

10. Molina JM, Ward D, Brar I, et al. Switching to fixed-dose bictegravir, emtricitabine, and tenofovir alafenamide from dolutegravir plus abacavir and lamivudine in virologically suppressed adults with HIV-1: 48 week results of a randomised, double-blind, multicentre, active-controlled, phase 3, non-inferiority trial. *Lancet HIV*. 2018;5(7):e357-e365. doi:10.1016/s2352-3018(18)30092-4

11. Briggs A, Sculpher M, Claxton K. *Decision Modelling for Health Economic Evaluation*. Oxford University Press; 2006.
